# Classifier for Activities with Variations†

**DOI:** 10.3390/s18103529

**Published:** 2018-10-18

**Authors:** Rabih Younes, Mark Jones, Thomas L. Martin

**Affiliations:** 1 Department of Electrical and Computer Engineering, Duke University, Durham, NC 27708, USA; 2 Department of Electrical and Computer Engineering, Virginia Tech, Blacksburg, VA 24061, USA; mtj@vt.edu (M.J.); tlmartin@vt.edu (T.L.M.)

**Keywords:** activity recognition, classifier design, complex activities, activities with variation, dataset

## Abstract

Most activity classifiers focus on recognizing application-specific activities that are mostly performed in a scripted manner, where there is very little room for variation within the activity. These classifiers are mainly good at recognizing short scripted activities that are performed in a specific way. In reality, especially when considering daily activities, humans perform complex activities in a variety of ways. In this work, we aim to make activity recognition more practical by proposing a novel approach to recognize complex heterogeneous activities that could be performed in a wide variety of ways. We collect data from 15 subjects performing eight complex activities and test our approach while analyzing it from different aspects. The results show the validity of our approach. They also show how it performs better than the state-of-the-art approaches that tried to recognize the same activities in a more controlled environment.

## 1. Introduction

Research in activity recognition has gained much attention in the past decade. From computer vision to wearable computing, research has led to developing products that rely on activity recognition approaches, such as the Microsoft Kinect, Fitbit, and others [1,2]. Although mostly used nowadays for fitness and gaming, research has shown that the importance of activity recognition goes far beyond that. Activity recognition could be used in health care, for accurate and transparent automatic ambulatory monitoring [3,4]. It could also be used to detect falls and health-related anomalies, which could save lives [5,6]. Activity recognition is also important to be able to understand different aspects of human behavior and emotions [7,8].

Even though recent studies have tried to address the needs of activity recognition systems and have managed to improve the accuracy for a wide variety of purposes, activity recognition systems are still far from ideal. One issue, in particular, that we are addressing in this work is the ability for systems to recognize complex daily activities that can occur in different variations. We are not only addressing variations within the same scripted activity that can occur between one person and another due to the difference in body structure or differences in the speed of performing a scripted activity; our approach goes beyond that to recognize wide variations of an activity that can occur when giving minimal guidance to the user. Traditional classifiers are usually good at recognizing simple, usually short, scripted activities [9,10]. By using the word “scripted”, we mean that the training procedure is very controlled and does not allow for much variation within the same activity. Having strict control over how activities are performed leads to the classifier being able to recognize only specific variations of the activity. Adding variations to an activity, such as performing a certain activity using the right hand versus using the left hand, would require retraining the activity recognition system with both exact variations for the system to be able to recognize both of them [9,11,12]. Being able to recognize such variations, and even more drastic ones, without the need to retrain the system for every possible combination of variations would be a leap toward making daily activity recognition more practical in a naturalistic environment (“in the wild”). Note that we are assuming that the complexity of the activity and its wide variations are the only factors impacting the complexity of the activity recognition problem. There are many other factors that go beyond the scope of this work that address other issues, such as the scalability of a recognition system and its ability to accommodate a larger number of activities, the recognition of transitions between activities and using them to improve recognition accuracy, and many others [13,14].

The novel aspect of our classifier, called “Classifier for Activities with Variations” (CAV), is breaking down activities into smaller parts. A vocabulary is formed using the commonly-found parts. The presence of those parts and transitions between them are then studied in order to recognize other variations of the same activity. Our method shows an improvement of 14% over the state-of-the-art.

The rest of the paper is organized as follows. We start by presenting the background of our work (Section 2). Afterwards, we explain the data collection procedure and our approach (Section 3). Then, we discuss the experimental procedure and the results (Section 4). Finally, we conclude the study and propose ideas for future work (Section 5).

## 2. Background

To understand our approach and contribution more clearly, we present some concepts that will be used later, and we contrast our work and purpose with other works in the field.

### 2.1. Pose, Motion and Activity

Previous works have defined some terms in activity recognition, such as gesture, action and activity. Some studies define gestures or atomic actions and then define activities as something formed by a group of gestures or actions, while other studies blend actions and activities [9,15].

Since no definition of “activity” is consistently adopted in the literature, we will use the following definitions throughout this paper, and we hope that future studies will use them as well:

- Pose—a pose is the position of the human body at one point in time. A unique body pose is defined by the unique angles of all body limbs with respect to each other, disregarding the location and orientation of the subject.
- Motion—a motion is a sequence of body poses throughout time. A motion is not bounded by a specific duration.
- Activity—an activity is a meaningful label that could be assigned to a motion.

### 2.2. Activity Recognition Dimensions

Activity recognition approaches have focused on different perspectives of activity recognition. In order to designate the activity recognition space that we are addressing, compared to other works, we come up with the following qualitative dimensions that could define the activity recognition space:

- System: the recognition system, which could be:
  1. User-dependent and sensor-dependent
  2. User-independent and sensor-dependent (or the less frequent case of user-dependent and sensor-independent)
  3. User-independent and sensor-independent
- Environment: the data collection environment, which could be:
  1. Fixed with tracked objects
  2. Fixed with objects not tracked
  3. In the wild
- Activities: the recognized activities, which could be:
  1. Unlabeled
  2. Labeled and strictly scripted
  3. Labeled and high-level scripted
- Activity segmentation: the segmentation that occurs in the inference procedure, which could be:
  1. Manual
  2. Automated
- Activity length: the length of the recognized activities, which could be:
  1. Known
  2. Bounded
  3. Unknown

In the above, we mean by “user-dependent systems” the recognition systems that only work for one user and need to be retrained for every new user. We mean by “sensor-dependent systems” the recognition systems that can only be used with one sensor domain. We mean by “fixed environment” a known environment that does not change while training the system or using it for inference. We mean by “unlabeled activities” the activities resulting from an unsupervised learning system where activity labels do not necessarily have a useful meaning that reflects actual human activities. We mean by “scripted activities” the activities whose training procedure is very controlled and does not allow for much variation within the same activity. We mean by “known” or “bounded” activity lengths the activities that have predetermined durations or durations that are defined to be within a certain interval.

**Figure 1:**
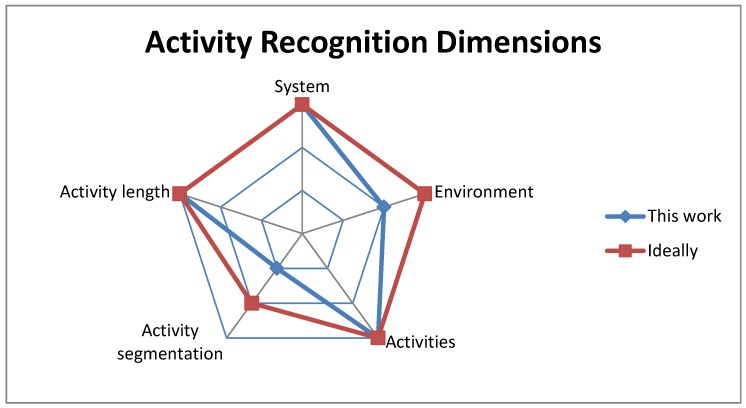
Activity recognition dimensions. “This work” designates the space that this paper is addressing, while “Ideally” designates the ideal space to be addressed.

The Kiviat chart in Figure 1 shows the space that we address in this paper. This space consists of a user-independent and sensor-independent system, a fixed environment where objects are not tracked, labeled and high-level scripted activities and manually-segmented activities that have an unknown (variable, but not bounded) length. This is in contrast to other studies that deal with sensor-dependent classifiers, strictly scripted activities, tracked objects, short activities or study activity spotting [9,16,17].

### 2.3. Activity Variations

Most activity classifiers, so far, have only been able to recognize specific ways of performing each activity to be classified. That is because classifiers are usually trained using a training set of examples consisting of users performing each activity in a certain way. Therefore, the developed activity classifier can only recognize those ways of performing the desired activities or other ways that are very close to the examples in the training set. Some examples of such activities are: walking, sitting, opening a specific type of door, etc. [9]. Some other classifiers can tolerate simple variations within the same activity, such as anthropometric variations and performance speed variations [10]. Anthropometric variations are differences between each subject’s movements through three-dimensional space, while performance speed variations are differences between each subject’s movements throughout time [18,19].

Using those activity classifiers, if it is necessary to detect multiple ways—not necessarily similar ways—of performing an activity, a training set of users performing each activity in each specific way is required. This makes the data collection and the training process very difficult and complicated, while not always being able to cover all the possible ways that an activity can be performed. This process gets even more complicated with complex activities that involve performing a sequence of steps, where the existence of every step and the order of those steps may vary. In fact, some previous works have studied the impact of multiple types of variations on the classification process and showed how complex variations have to be treated as different activities when using traditional classifiers [11,12]. In that case, instead of having to collect data and train the classifier using *N* activity sets, we would have to do that using N×V activity sets. *N* designates the number of activities, while *V* designates the average number of variations per activity.

To clarify this aspect, we give the following example. In the case of “using a computer”, there is not one specific way to perform this activity. This complex activity may or may not involve steps such as typing on a keyboard, using a mouse, sitting, sitting while crossing feet, etc. The order and length of those steps also vary. Recognizing such activities is not possible when using traditional classifiers. A new type of classification is needed to take into account the properties of such complex activities. In addition, when recognizing users’ daily activities, it would be better to know more meaningful and useful information about their routine. For example, if the user is cleaning a table, we would like to recognize this activity as it is, instead of recognizing that the user grabbed something with his/her hand then walked for a while (around the table) while performing some “Karate Kid” moves (i.e., rotating hand movements while cleaning the table).

## 3. Materials and Methods

### 3.1. Data Collection

We collected a dataset, called $Dataset_{variations}$, that consists of data corresponding to eight complex heterogeneous activities of daily living, involving movements of all limb segments in sitting and standing positions. The Oxford Dictionary (currently used as Google’s dictionary) defines “complex” as “consisting of many different and connected parts” [20]. In the context of activities, the same definition applies to a “complex activity”. The same dictionary defines “heterogeneous” as “diverse in character or content” [21]. In this context, “heterogeneous activities” designates activities having wide amounts of variations between different samples of the same activity. According to the above definitions, the following are the corresponding mathematical definitions for “complexity” and “variation”:

(1) $$Complexity=\sum_{s=1:length_{A}}Dist\left(A,A_{shifted}\left(s\right)\right)$$

and

(2) $$Variation=\bar{Dist(A_{i},A_{j})}$$

where *A* designates an activity, $A_{shifted}\left(s\right)$ designates a shifted version of the activity where the activity frames are rotated by *s* seconds, Dist(x,y) computes the Euclidean distance between *x* and *y* and $\bar{Dist(A_{i},A_{j})}$ computes the average Euclidean distance between all combinations of normalized activities in a set. In other words, an activity’s Complexity is a value that represents the autocorrelation of the activity, while a set’s Variation is a value that represents the correlation between the activities in the set.

In this dataset, 15 subjects performed each activity once, while data were being captured by the Qualisys video motion capture system. The Qualisys motion capture system was chosen due to its high accuracy, in order to eliminate factors that could cause data inaccuracy. The main purpose of this dataset is to have complex heterogeneous activities of daily living that allow for a wide range of variations within each activity, thus enabling this dataset to be used for algorithms trying to classify more naturalistic activities.

Chosen activities consisted of complex activities that other recent studies had difficulty recognizing in a scripted and controlled environment [10,22,23,24]. For example, in [10], the activity of using a computer while sitting scored an accuracy of 47.5%. This activity’s accuracy stands out compared to other activities that they recognized with much higher accuracy rates, which were typically in the range of 60–100%. In addition, all chosen activities have a wide range of significantly distinct variations. The scripts used for this dataset’s activities are high-level scripts, allowing for a high amount of freedom and variations. Before subjects performed each activity, they were always reminded to perform it as they usually do in their natural setting. Additionally, every participant was asked to perform each activity only once, so that participants would not have to fake repetitions of the activity or add bias to the data by repeating the same steps multiple times.

In the following, $Dataset_{variations}$’s activities are presented along with their environmental settings:

1. Eating:
  Before recording starts: The subject chooses what to eat and which utensils to use among an available variety of food and utensils. The subject then sits at a table with his/her food and utensils in front of them.
  While recording: The subject eats their food.
2. Taking medication:
  Before recording starts: The subject stands in front of a table that has three types of medication containers—two types of pill bottles that open differently and a pill packet (actual pills are not used; candy is used instead)—and a cup of water.
  While recording: The subject chooses one medication and takes one “pill”.
3. Brushing teeth:
  Before recording starts: The subject stands in front of a table that has a toothbrush, toothpaste and a cup of water.
  While recording: The subject brushes his/her teeth. He/she may clean his/her mouth using the cup of water, and he/she may also spit in it.
4. Mopping:
  Before recording starts: The subject chooses a mop among two available types. The subject chooses an empty (no furniture) area to be mopped. The subject stands (holding the mop) at a chosen starting point in the designated mopping area.
  While recording: The subject mops the entire designated mopping area.
5. Using a computer:
  Before recording starts: The subject sits at a table with his/her laptop in front of him/her. A mouse is provided in case he/she prefers using it instead of the laptop’s touchpad. The laptop is up and running.
  While recording: The subject opens his/her email and looks for an email from the researcher asking him/her how his/her day was. He/she reads the email and then replies to it.
6. Writing:
  Before recording starts: The subject sits at a table with two types of notebooks and two types of pens in front of him/her. The subject chooses a notebook and a pen to use.
  While recording: The subject writes a few lines about his/her day in the chosen notebook.
7. Making a cell phone call:
  Before recording starts: The subject stands while having his/her cell phone in one of his/her pockets.
  While recording: The subject picks up his/her phone, calls the researcher, waits for the phone to ring two or three times, hangs up and puts the phone back in his/her pocket.
8. Driving:
  Before recording starts: The subject sits in front of a driving gaming system consisting of a computer, a steering wheel, a transmission and pedals. A driving game is loaded on the computer.
  While recording: The subject drives to a randomly chosen location in the game.

While ignoring basic variations, such as performance speed variations, the following are some of the various ways different subjects performed the activities:

- For eating: Sitting at the table with different postures. Knee angles for each leg varying between 70 and 120 degrees. Using a spoon, fork or chopsticks. Using right or left hand. Having the other hand on the table or on the leg. Different leaning angles while approaching food.
- For taking medication: The choice of pill bottle or packet. Picking the pill bottle/packet using the right or left hand. Easily getting the pill out or struggling while extracting only one pill out of the bottle. Using right or left hand to take the pill. Using right or left hand to drink the water. Taking the pill before drinking the water, or vice versa.
- For brushing teeth: Picking up the toothbrush and toothpaste with the right or left hand. Using toothpaste or not using it. Different techniques of brushing teeth. Picking up the cup of water with the right or left hand. Cleaning mouth with water or not. Spitting once or multiple times.
- For mopping: The choice of mop type. Putting the right or left hand on top. Switching between hands or not. Different leaning angles, ranging between 10 and 60 degrees. Performing linear or circular mopping movements. Starting/ending at different locations and traveling different paths in between.
- For using a computer: Different knee angles for each leg. Different leaning angles. Using the mouse or not. Using the mouse only at the beginning or using it intermittently throughout the activity. Using the mouse with the right or left hand.
- For writing: Choice of notebook and pen types and consequently the way subjects open/close them. Opening the book and the pen with the right or left hand. Putting the notebook and writing at different angles. Writing with the right or left hand. Putting the other hand on the notebook, the table or the leg. Different leaning angles. Different knee angles for each leg.
- For making a cell phone call: Having the cell phone in the right or left, front or back pocket. Using the right or left hand to pick the phone up, tap on the screen, putting it to the ear, hanging up and putting it back in the pocket. Using one or both hands when typing on the phone. Having to tap the numbers of the phone number or having the number already saved on the phone. Walking/moving while making the call or not.
- For driving: Different knee angles for both legs. Different ways of using the steering wheel. Using the transmission or not.

**Figure 2:**
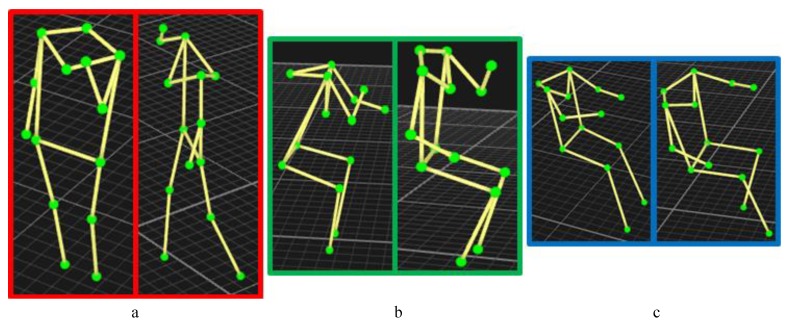
Screenshots of data from the Qualisys motion capture system showing some variations in the way different subjects perform the same activity. (**a**) shows variations in making a cell phone call; (**b**) shows variations in eating; and (**c**) shows variations in driving.

Some of the mentioned variations are shown in Figure 2. The nodes in Figure 2 (green spots) represent the reflective markers that were placed on each subject’s body. (a) shows variations within the making a cell phone call activity; it shows the subject on the left side holding the phone with his/her left hand while standing, while the subject on the right side holds the phone with his/her right hand and walks while making the call. (b) shows variations within the eating activity; it shows the subject on the left side eating using his/her right hand while putting his/her left hand on the table, while the subject on the right side eats with his/her left hand while putting his/her right hand on his/her right leg and having a different posture (back and legs angles) than the subject on the left side. (c) shows variations within the driving activity; it shows the subject on the left side driving with both hands on the wheel while stretching his/her left leg, while the subject on the right side drives using one hand while having the other on the transmission and his/her left leg at a different angle from the subject on the left side.

In addition to the above variations, subjects also showed some short variations that often occurred while performing most activities, such as randomly moving the legs while performing an activity, scratching the face and other movements that did not relate to the high-level scripts.

**Figure 3:**
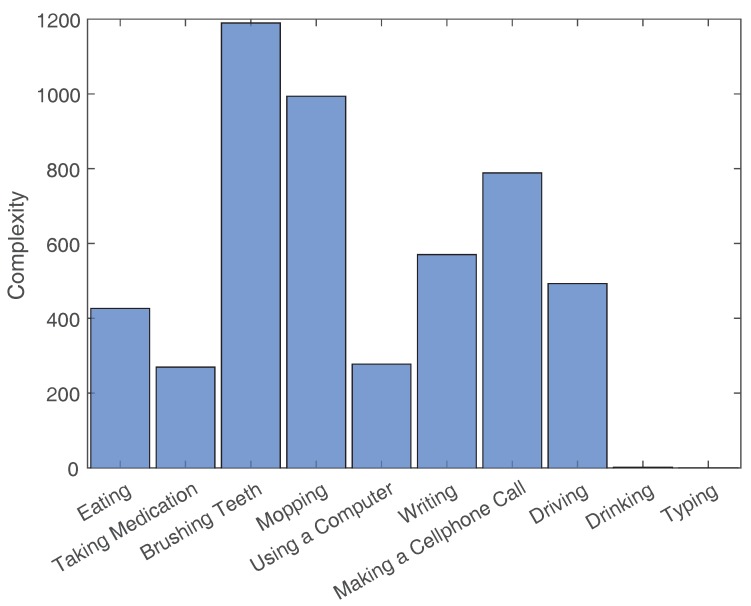
Average Complexity values for the activities of $Dataset_{variations}$ and two other simple activities (i.e., Drinking and Typing).

**Figure 4:**
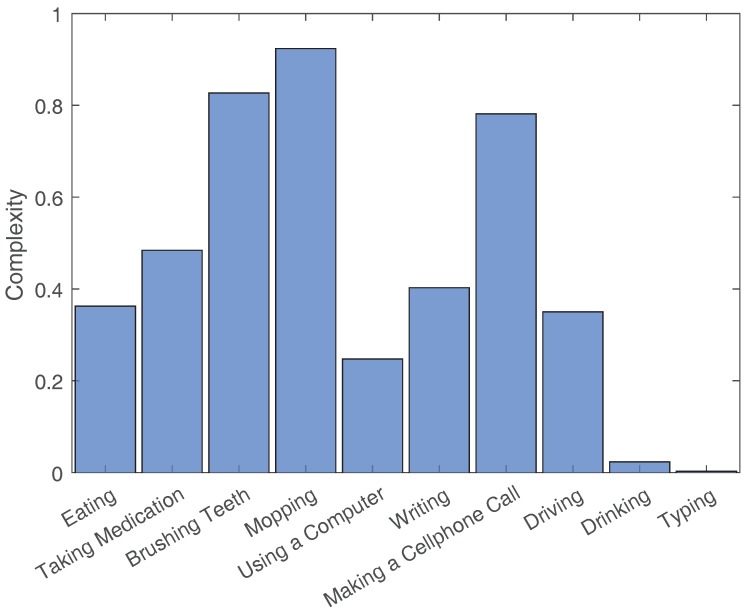
Complexity values normalized by activity length.

**Figure 5:**
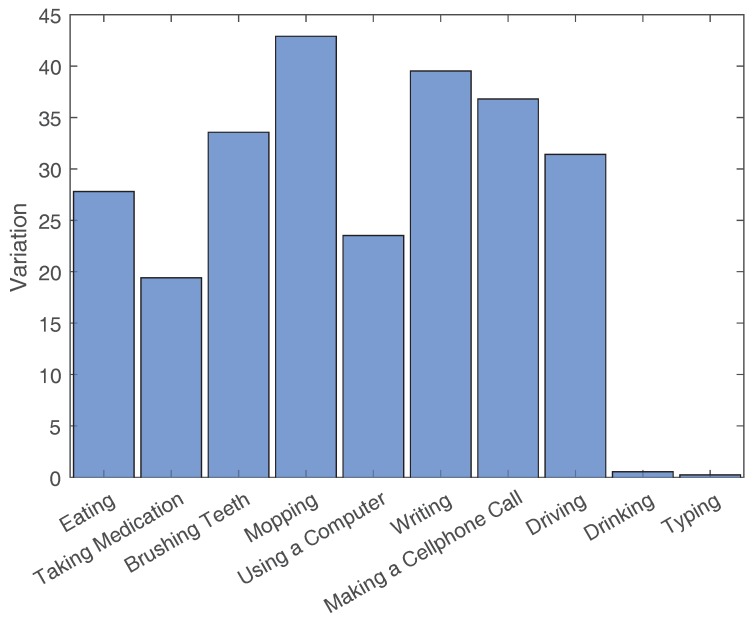
Variation values for the activities of $Dataset_{variations}$ and two other simple activities (i.e., Drinking and Typing).

**Figure 6:**
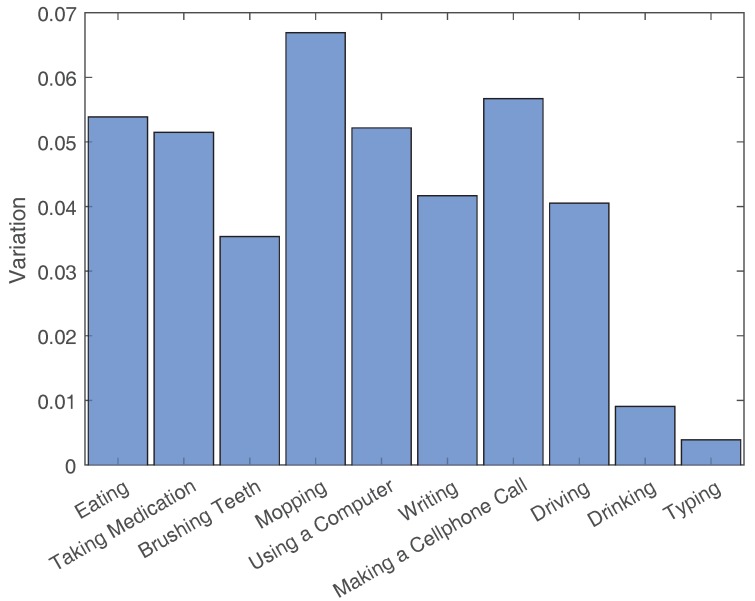
Variation values normalized by activity length.

The average Complexity values and the Variation values for each activity’s example set are shown in Figure 3, Figure 4, Figure 5 and Figure 6. To contrast other simpler activities, the values for two simple activities are added to the eight activities of this dataset. The first activity is Drinking, which consists of drinking water from a cup that is placed on a table. The second activity is Typing, which consists of typing on a keyboard placed on a table while sitting in a chair.

### 3.2. Classifier for Activities with Variations

The main goal of CAV is to divide the complex activity into smaller chunks, then use the extracted information about their presence and order to make inferences. As with most classification approaches, the two main phases of CAV are the training phase and the inference phase. The training phase is when representations of activities are created based on a training dataset, while the inference phase is when another dataset’s observations are classified based on the activity representations created during training.

The following sections discuss the training and inference phases of the developed approach. Note that, after taking labeled datasets as input, both the training phase and inference phase are fully automated.

#### 3.2.1. Training

The training set is given as a set of labeled activities. As explained in Section 3.1, each activity is represented by a sequence of body poses throughout time. Each pose is a nine-segment body model in quaternion space. Note that CAV can work with data originating from different sensor domains as long as the data (quaternions, angles, accelerations, positions, etc.) of the body limbs are given with respect to the body, not something else in the environment.

**Figure 7:**
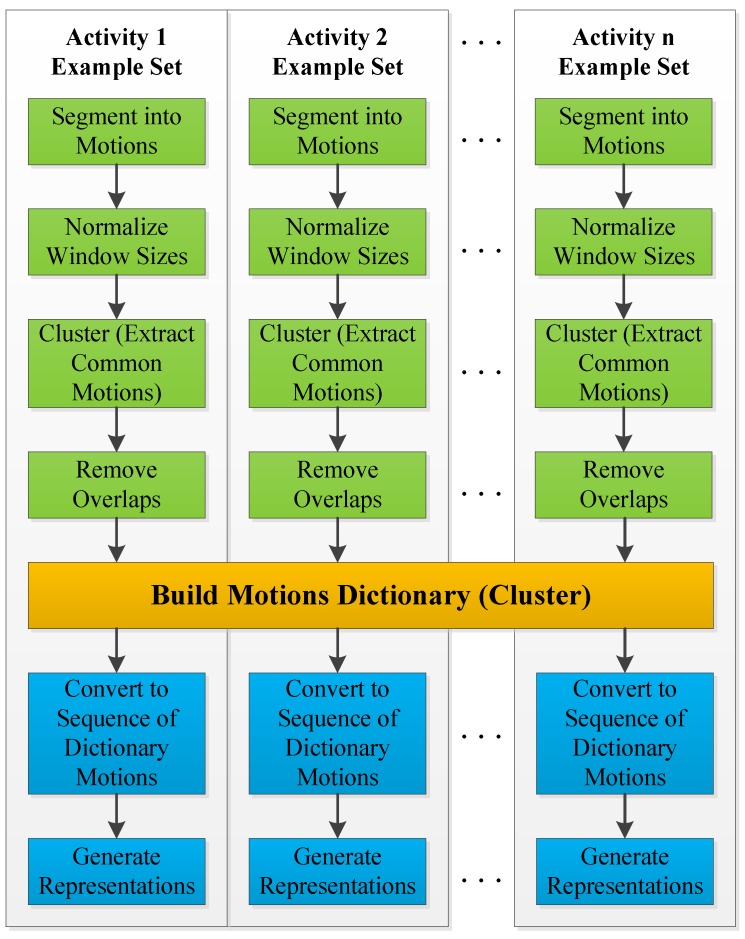
Flowchart of the training phase.

The main steps of the training phase consist of extracting common motions from all examples in the training set in order to build a dictionary of motions, then generating activity representations for each example to be used during inference. These steps are summarized in the flowchart of Figure 7 and are explained in the following.

**Figure 8:**
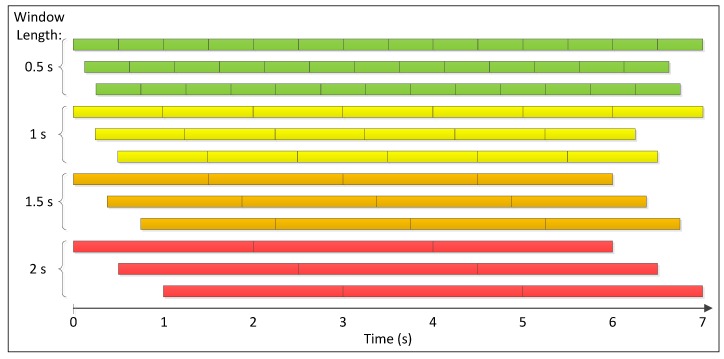
An example showing all windows obtained from a 7-second activity; starting from 0.5-second window segments (top) to the 2-second window segments (bottom), showing the three repetitions (increment of 0%, 25% and 50% of the window size) for each window size.

**Figure 9:**
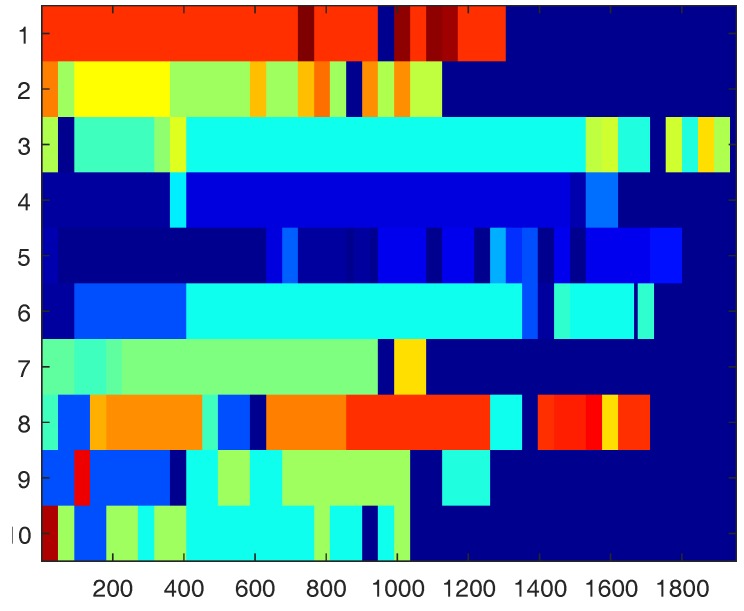
Motion clustering results for brushing teeth. The x-axis designates frame numbers (at 30 Hz). The y-axis designates participants’ IDs. Motions belonging to the same cluster have the same color. The plot shows how different subjects perform the same activity very differently while many motions repeat among multiple subjects [26].

- Segment motions:
  Each example in the training set is first segmented into windows using four sizes that represent the lengths of 0.5, 1, 1.5 and 2 s. The four window lengths are chosen after studying the lengths of repeated motions and concluding that they usually vary within the chosen range. The segmentation process is repeated three times with segmentation increments of 0%, 25% and 50% of the window size; i.e., the starting points of all windows are shifted by 0%, 25% and 50% of the window size. An example of all resulting windows is shown in Figure 8. Windows longer than 0.5 s are sub-sampled, so that all extracted windows are normalized and have the same size.
- Extract common motions:
  For each activity, all extracted windows are clustered using k-means clustering [25]. The distance metric used for k-means is Euclidean distance. Clusters containing a low number of windows (five windows or less) are dropped since only motions that often repeat are of interest. Afterward, overlapping windows are examined. Windows with larger sizes are kept, while their overlapping windows are removed. Figure 9 shows the results after these steps are performed on the brushing teeth activity.
- Build motions dictionary:
  This step consists of creating a dictionary of motions. First, cluster centers for all activities are clustered using k-means clustering. This second clustering is realized to make sure that motions in the dictionary are distinct since similar motions could exist between two or more activities. The motions dictionary is filled with the new cluster centers that form its vocabulary. Note that, at every step, the algorithm keeps track of where the cluster centers originated. This tracking enables it to get back to every motion in the original examples and label it with the correct dictionary motion ID. The dictionary motion ID is a unique number that identifies each motion in the dictionary.
- Generate activity representations:
  After converting each example in the training dataset to a sequence of dictionary motion IDs, two features that can be used to represent each example are extracted. The first feature is a histogram that contains the frequency of occurrence of each dictionary motion in the example. The second feature is a transition matrix containing normalized transition probabilities between motions for that example.
  To have better-weighted histograms that account for the importance of each motion, log-normalized tf-idf (term frequency-inverse document frequency) is used on all histograms, and a stop list is applied to ignore the top 5% most often occurring motions [27,28].

#### 3.2.2. Inference

After training the classifier, the inference is performed subsequently in the following manner.

Segmented observations are given to the classifier; each observation consists of only one activity. CAV cannot recognize the presence of a known activity within a stream of data; it cannot automatically detect where the activity starts and where it ends. CAV does not address automatic activity spotting since activity spotting and segmentation algorithms are out of this work’s scope; they are thoroughly studied in other works [29,30,31]. Each observation in the inference dataset is traversed using the four window sizes, as discussed in Section 3.2.1. Each window is then matched, if possible, to a motion that exists in the motion dictionary. When a window matches, the algorithm skips to the adjacent window, without having to go through each window increment. As a result, each observation is converted to a sequence of dictionary motions (motion IDs). Afterward, a histogram of motions is created for each observation while considering the same tf-idf normalization and stop list used in Section 3.2.1.

Each observation is compared to all activity representations resulting from the training phase. While comparing, two metrics are extracted:

- histDiff: the difference between the observation’s motions histogram and the potential match’s motions histogram.
- seqProb: the observed motions sequence probability.

histDiff is calculated using Euclidean distance. seqProb is calculated using the equation:

(3) $$seqProb=\mathrm{Pr}\left(m_{1}\right)\times \prod_{i=2}^{n}\mathrm{Pr}\left(m_{i}|m_{i-1}\right)$$

where *n* is the number of motions in the observation, $m_{i}$ is the motion (motion ID) at *i* in the observation’s motion sequence and $\mathrm{Pr}(m_{i}|m_{i-1})$ is the probability of transitioning from $m_{i-1}$ to $m_{i}$, extracted from the potential match’s transition matrix.

After computing histDiff and seqProb, they are both aggregated to form one cost measure, which is:

(4) $$cost=\frac{histDiff}{seqProb}$$

The resulting costs from all examples of each activity are averaged to form an activity cost; one cost associated with each activity. The observation is then matched to the activity yielding the lowest activity cost.

**Figure 10:**
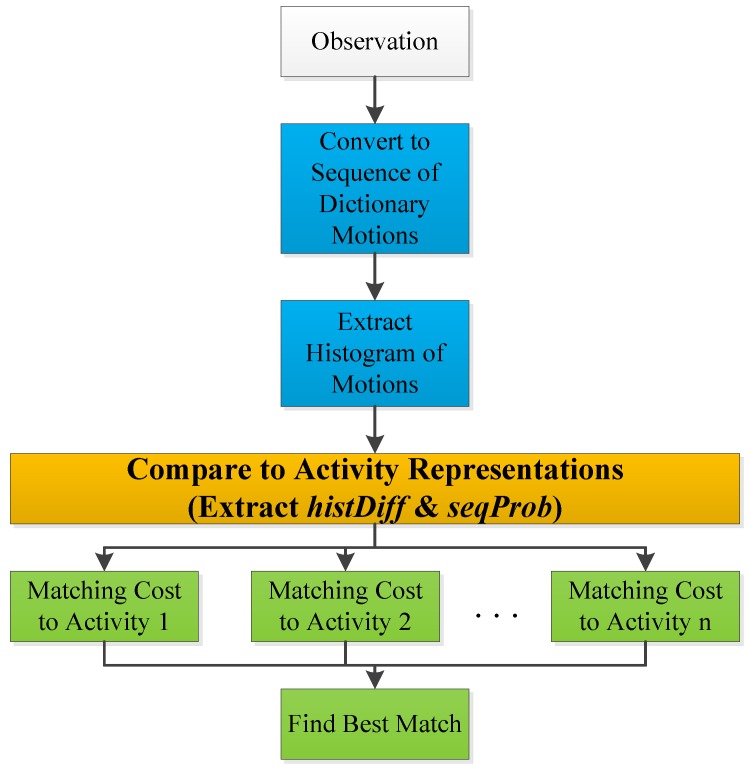
Flowchart of the inference phase.

These above steps are summarized in the flowchart of Figure 10 and are explained in the following.

## 4. Results and Discussion

Using the data of $Dataset_{variations}$, CAV was evaluated while performing the following analyses.

### 4.1. Cross-Validation

First, a leave-one-out cross-validation was performed using all the examples of the dataset ($Analysis_{1}$). This analysis shows the true discriminating capabilities of the algorithm. The leave-one-out cross-validation rotates throughout the whole dataset while classifying—at each iteration—one example against the remaining examples, which are used for training [32,33,34]. This means that, at every iteration, our training set had the 14 remaining iterations of the activity whose sample was used for inference and the remaining 15 samples of each of the other seven activities. The results are shown in Table 1.

The results yielded an overall accuracy of 93.33%, or 7.46-times better than chance (chance = $\frac{1}{8}$). Fifty percent of the activities had a 100% accuracy, while the rest had an accuracy of 80% and above. Misclassified activities were confused with those that they shared with a large number of motions. For example, the activities of driving, usingComputer and eating all consisted of users performing motions while sitting and having their arms in front of them. Therefore, this similarity resulted in some driving and eating examples being confused with usingComputer. The same analysis applies to the other set of activities that got a little confused. The activities of cellphoneCall, takingMeds and brushingTeeth all contained motions involving standing up and having arms moving back and forth from near the user’s face to a table-height position. This similarity resulted in some takingMeds and brushingTeeth examples being confused with cellphoneCall.

### 4.2. Narrowing the Training Set’s Variations

This analysis ($Analysis_{2}$) concerns testing CAV’s capability of generalizing to new activity variations with which it was not trained. For this purpose, two-thirds of each activity’s examples that had the narrowest range of variation—an average variation value of 22 compared to 32 of the whole dataset (see Figure 5)—were selected. These examples were used as the training dataset, while the rest of the examples served as the inference dataset. The results of this analysis are shown in Table 2.

The results yielded an overall accuracy of 60%, or 4.8-times better than chance. The lowest accuracy obtained per activity was 40%, while the highest was 100%. Similar to Section 4.1, misclassified activities were confused with those that they shared with a large number of motions. Going from $Analysis_{1}$ to $Analysis_{2}$, the mopping activity conserved its accuracy of 100%, thus showing how a small amount of variation was enough to train for many different variations of mopping. On the other hand, the accuracy of other activities dropped because, when narrowing the variation range, these activities got closer to others in the solution space, which made them get confused with one another. For example, looking at the most extreme case of the usingComputer activity, its accuracy dropped from 100% in $Analysis_{1}$ (see Table 1) to 40% in $Analysis_{2}$ (see Table 2). That is because, when the range of variation in the training sets got much narrower, the activity representations of usingComputer and eating became very similar to an extent that they got confused with one another. Both these activities involved someone sitting in a chair while usually leaning forward and having both arms near the surface of the table most of the time. In fact, we can see some of that confusion appear in the results of $Analysis_{1}$ (see Table 1) when the eating activity showed a little confusion (6.67%) with usingComputer.

The relatively high average accuracy of the results of $Analysis_{2}$ suggests that CAV might be able to recognize many more variations of an activity than the variations with which it was trained. Using a training example set that has a slightly larger number of variations within each activity, much higher accuracy rates could be reached while detecting new variations. This experiment was realized, and its results are shown in Table 3.

In the latter experiment ($Analysis_{3}$), 10 out of the 15 participants (i.e., two thirds) were randomly picked to form the training dataset, while the rest formed the inference dataset. Equal gender proportions were maintained between the training and inference datasets. This process was repeated 20 times, and the results were averaged. The results (see Table 3) yielded an overall accuracy of 86.13%, or about seven-times better than chance. The mopping activity maintained the 100% accuracy, and the rest of the activities had accuracy rates ranging between of 76% and 97%. Misclassified activities were still only confused with those that they shared with a large number of motions. These results show a significant improvement—from 60%–86.13%—that occurred when only adding a small number of variations (average variation = 25) to the training dataset. This shows that CAV only needs to be trained with a small number of variations of an activity in order to be able to recognize its other variations with a high accuracy.

### 4.3. Comparing to Previous Methods

In this analysis, CAV is compared to other state-of-the-art methods in the field of wearable activity classification. Three works were chosen from the literature to compare to CAV, each of them having an overlap with CAV’s methodology or purpose. These works were:

1. Zinnen’s classifier [9]: This is the state-of-the-art work in wearable full-body activity recognition. It performs better than any other full-body user-independent activity classifier and is considered the state-of-the-art in multiple recent works [35,36,37,38]. It relies on an abstract body model that makes it user-independent and capable of being used with data from multiple sensor domains. This work was chosen because it had most of the characteristics that are needed in an ideal wearable activity classifier, as described in [39].
2. Berlin’s classifier [40]: This work extracts “motifs” to be used in order to detect one simple fixed-length activity within a background of non-activity data. Motifs are reoccurring sequences of signal-oriented time series’ symbols that have fixed lengths. This work was chosen because it used a similar concept to CAV that relied on splitting the activities into smaller chunks.
3. Peng’s classifier [41]: This is a more recent work that tried to classify complex activities by applying a method similar to what was used in Berlin’s classifier, but where smaller chunks—“actions”—were extracted and aggregated in a different way.

Note that none of the above methods have previously been compared using the same dataset. For each work, the proposed algorithm was implemented and tested with the same dataset used to test CAV (i.e., $Dataset_{variations}$).

Zinnen’s classifier was implemented as it was described in their work while using the algorithm that did not consider location features since the dataset used did not provide location information. Testing Zinnen’s classifier yielded an accuracy of only 37.5%. This classifier was also tested while using short motion sets as its activity sets. These sets consisted of motions that were clustered together when the common motions were extracted from activities using CAV. The motions in the sets were short motions that were very similar and did not have many variations within each set. The results obtained by testing the motion sets on Zinnen’s classifier yielded an increased accuracy that matched their paper’s results. This contrast shown in the results means that CAV performed much better than Zinnen’s classifier when it came to recognizing complex naturalistic activities that could have variations and/or relatively long durations.

Berlin’s classifier was implemented as it was detailed in their work, and testing it yielded an accuracy of 58.6%. The main reasons behind the improved accuracy of CAV over Berlin’s classifier were:

- Berlin’s classifier used only a bag-of-words approach for the motifs, while CAV used information about the transitions between the motions in addition to the bag-of-words approach.
- The motifs extracted in Berlin’s classifier were reoccurring sequences of symbols that had fixed lengths, while CAV’s motions can vary in length.

Peng’s classifier was implemented as it was described in their work while leaving out the part that considered features derived from physiological signals, since these measurements were not available in the dataset used. Testing Peng’s classifier yielded an accuracy of 65.8%. The main reasons behind the improved accuracy of CAV over Peng’s classifier was similar to those of the second algorithm: using fixed-length windows and ignoring the transitions between the words/topics. Using some advanced statistical analysis in Peng’s method, which included extracting topics from words and computing a statistical feature vector to use for matching, gave this method an advantage over Berlin’s method; therefore confirming the results obtained in [41] when comparing Peng’s classifier to Berlin’s.

To make sure that CAV outperformed the three methods above with statistical significance, a paired *t*-test was performed with each method. The test took pairs that consisted of each subject’s accuracy in both CAV and the method to which it was compared. A value of α=5% was used for the tests. The tests were successful, and the null hypothesis was rejected, which means that the obtained results’ data were statistically significant; thus, CAV significantly outperformed the three methods.

It is important to note that, for each of the chosen activities, CAV outperformed recent approaches that have tried to recognize some of the activities used in $Dataset_{variations}$ in a more controlled environment [10,22,23,24]; i.e., CAV’s recognition accuracy for those activities was higher than theirs. For example, the usingComputer activity yields an accuracy of 100% using CAV, while only scoring 47.5% in [10]. Furthermore, in [10], this activity was scripted, not similar to other activities in their dataset, and scored an accuracy that was much lower than their obtained overall accuracy rate. There was not a direct comparison to these approaches—i.e., implementing their classifiers and testing them using $Dataset_{variations}$—because they were not the state-of-the-art in recognizing complex/varying activities; they focused on strictly-scripted activities. For example, in [10], the purpose was to create a user-independent and sensor-independent activity classifier. Their target data consisted of repeated strictly-scripted activities—no freedom for the user to perform the activity differently or interact with other objects—that were performed using different subjects and two different sensor domains.

Besides showing CAV’s superiority when it comes to classifying complex activities with variations, the results obtained in this section presented some key techniques that can be used when trying to classify such activities, such as:

- Using smaller chunks of the activity: algorithms using features for smaller parts of the complex activity were shown to yield better results than others that used the whole activity as it was.
- Extracting the smaller chunks in a similar way to CAV, which allowed variation in length: taking into account the variations in the duration of a motion was shown to yield better results than other methods that used fixed-length motions/motifs/actions.
- Using temporal relationships between the small chunks: considering both the presence of the motions and their order within the activity was shown to yield better results than other methods that relied only on the presence of motions/motifs/actions within the activity.

## 5. Conclusions and Future Work

This work introduces a new classifier, CAV, that enables recognizing complex activities that could be performed in a wide variety of ways. It was shown that, by breaking down complex activities into motions and using a dictionary of motions, the proposed method can successfully recognize complex variations of activities with an accuracy of 93.33%. It was also shown that CAV can accurately recognize a large number of complex variations of activities after only learning a small subset of these variations. CAV constitutes a step farther toward being able to recognize complex activities “in the wild” while using a small sample of their variations. As mentioned earlier, CAV’s main limitations consist of having segmented activities and having data representing movements of body limb segments with respect to the body itself, not the external environment.

Future work will focus on optimizing the extraction of motions that form the dictionary, optimizing the cost function, integrating activity spotting and publishing a dataset for benchmarking classifiers in recognizing complex varying activities.

## Figures and Tables

**Table 1:** Confusion matrix for $Analysis_{1}$. The value in each cell represents that the percentage that the row’s activity observation is matched to the column’s activity representation.

	Mopping	CellphoneCall	TakingMeds	BrushingTeeth	Driving	UsingComputer	Eating	Writing
**mopping**	**100**	0	0	0	0	0	0	0
**cellphoneCall**	0	**100**	0	0	0	0	0	0
**takingMeds**	0	13.33	**86.67**	0	0	0	0	0
**brushingTeeth**	0	20	0	**80**	0	0	0	0
**driving**	0	0	0	0	**86.67**	13.33	0	0
**usingComputer**	0	0	0	0	0	**100**	0	0
**eating**	0	0	0	0	0	6.67	**93.33**	0
**writing**	0	0	0	0	0	0	0	**100**

**Table 2:** Confusion matrix for $Analysis_{2}$. The value in each cell represents the percentage that the row’s activity observation is matched to the column’s activity representation.

	Mopping	CellphoneCall	TakingMeds	BrushingTeeth	Driving	UsingComputer	Eating	Writing
**mopping**	**100**	0	0	0	0	0	0	0
**cellphoneCall**	0	**80**	0	20	0	0	0	0
**takingMeds**	20	20	**40**	20	0	0	0	0
**brushingTeeth**	0	40	0	**60**	0	0	0	0
**driving**	0	0	0	0	**60**	40	0	0
**usingComputer**	0	0	0	0	0	**40**	60	0
**eating**	0	0	0	0	0	20	**40**	40
**writing**	0	0	0	0	0	40	0	**60**

**Table 3:** Confusion matrix for $Analysis_{3}$. The value in each cell represents the percentage that the row’s activity observation is matched to the column’s activity representation.

	Mopping	CellphoneCall	TakingMeds	BrushingTeeth	Driving	UsingComputer	Eating	Writing
**mopping**	**100**	0	0	0	0	0	0	0
**cellphoneCall**	0	**97**	0	3	0	0	0	0
**takingMeds**	0	14	**82**	4	0	0	0	0
**brushingTeeth**	0	23	0	**77**	0	0	0	0
**driving**	0	0	0	0	**78**	22	0	0
**usingComputer**	0	0	0	0	0	**83**	17	0
**eating**	0	0	0	0	0	13	**76**	11
**writing**	0	0	0	0	0	4	0	**96**
